# Reduced Orbitofrontal Gray Matter Concentration as a Marker of Premorbid Childhood Trauma in Cocaine Use Disorder

**DOI:** 10.3389/fnhum.2018.00051

**Published:** 2018-02-15

**Authors:** Keren Bachi, Muhammad A. Parvaz, Scott J. Moeller, Gabriela Gan, Anna Zilverstand, Rita Z. Goldstein, Nelly Alia-Klein

**Affiliations:** ^1^Departments of Psychiatry and Neuroscience, Icahn School of Medicine at Mount Sinai, New York, NY, United States; ^2^Department of Psychiatry, Stony Brook University School of Medicine, Stony Brook, NY, United States; ^3^Department of Psychiatry and Psychotherapy, Central Institute of Mental Health, Medical Faculty Mannheim, Heidelberg University, Mannheim, Germany

**Keywords:** cocaine dependence, childhood maltreatment, gray matter, voxel-based morphometry, addiction

## Abstract

**Background**: Childhood trauma affects neurodevelopment and promotes vulnerability to impaired constraint, depression, and addiction. Reduced gray matter concentration (GMC) in the mesocorticolimbic regions implicated in reward processing and cognitive control may be an underlying substrate, as documented separately in addiction and for childhood trauma. The purpose of this study was to understand the contribution of childhood maltreatment to GMC effects in individuals with cocaine use disorder.

**Methods**: Individuals with cocaine use disorder were partitioned into groups of low vs. high childhood trauma based on median split of the total score of the Childhood Trauma Questionnaire (CTQ; CUD-L, *N* = 23; CUD-H, *N* = 24) and compared with age, race, and gender matched healthy controls with low trauma (*N* = 29). GMC was obtained using voxel-based morphometry applied to T1-weighted MRI scans. Drug use, depression and constraint were assessed with standardized instruments.

**Results**: Whole-brain group comparisons showed reduced GMC in the right lateral orbitofrontal cortex (OFC) in CUD-H as compared with controls (cluster-level *p*_FWE-corr_ < 0.001) and CUD-L (cluster-level *p*_FWE-corr_ = 0.035); there were no significant differences between CUD-L and controls. A hierarchical regression analysis across both CUD groups revealed that childhood trauma, but not demographics and drug use, and beyond constraint and depression, accounted for 37.7% of the variance in the GMC in the right lateral OFC (*p* < 0.001).

**Conclusions**: Beyond other contributing factors, childhood trauma predicted GMC reductions in the OFC in individuals with cocaine use disorder. These findings underscore a link between premorbid environmental stress and morphological integrity of a brain region central for behaviors underlying drug addiction. These results further highlight the importance of accounting for childhood trauma, potentially as a factor predisposing to addiction, when examining and interpreting neural alterations in cocaine addicted individuals.

## Introduction

The child maltreatment-morbidity connection makes up for a large fraction of the global burden of all disease and premature mortality (Shalev et al., 2013, 2016; Chen et al., 2016). For example, childhood maltreatment (and associated adversity) accounts for 64% of the population-attributed risk for addiction to illicit drugs (Dube et al., 2003), potentially predisposing vulnerable individuals to substance use disorders and comorbid psychopathology. Specifically, childhood maltreatment increased the likelihood of lifetime substance use disorders, including the initiation of substance use especially during early adolescence (Dube et al., 2003; Shin et al., 2010). Childhood maltreatment has also been implicated in greater substance-cue reactivity and enhanced substance-use symptom severity inclusive of interpersonal and substance-related financial problems and risk of relapse (Westermeyer et al., 2001; Schumacher et al., 2006; Van Dam et al., 2014).

The potential impact of childhood maltreatment on substance use are elucidated via examination of how early exposures to social-relational stress may affect neurodevelopment in prefrontal cortical regions (De Bellis et al., 2002; Hanson et al., 2010; Hart and Rubia, 2012; De Bellis and Zisk, 2014; Paquola et al., 2016; Teicher et al., 2016) crucial for the development of highest-order cognitive abilities (Arnsten, 2009). Exposure to even brief periods of intense stress causes significant structural remodeling within the rodent prefrontal cortex (see Holmes and Wellman, 2009 for review). This structural remodeling involves impairments in orbitofrontal cortex (OFC)-dependent cognitive functions via excessive stimulation of dopaminergic and noradrenergic receptors (see Arnsten, 2009 for review), thereby enhancing drug self-administration (see Miczek et al., 2004; Sinha, 2008 for reviews). Children who have experienced early maternal deprivation exhibit an accelerated development in prefrontal cortical-amygdala connectivity as measured with an fMRI task of viewing emotional faces and analyzed with a psychophysiological interaction analysis (Gee et al., 2013). Previous reports suggest that this accelerated brain development in humans parallels that in rodents (McEwen, 2008; Callaghan and Richardson, 2011) and that it is interpreted to reflect an ontogenetic adaptation to adversity before adulthood (Anisman et al., 1998; de Kloet and Oitzl, 2003; Callaghan and Richardson, 2011). Such neurodevelopmental structural remodeling in mesocorticostriatal regions and specifically in the OFC, may be key in development of substance use disorders since these regions mediate behaviors of cognitive and impulse control and reward processing (e.g., enhanced drug over natural reward salience concomitant with a decrease in inhibitory control), as highlighted by the Impaired Response Inhibition and Salience Attribution (iRISA) model (Goldstein and Volkow, 2002, 2011).

Studies of adolescents and adults without psychopathology reported widespread reductions in gray matter (GM) volumes in several brain regions in childhood trauma victims (e.g., Tomoda et al., 2009: visual association cortices; Edmiston et al., 2011: corticostriatal-limbic regions; Cohen et al., 2006: anterior cingulate cortex and caudate nuclei; Thomaes et al., 2010: anterior cingulated cortex, OFC and hippocampus; Rinne-Albers et al., 2017: anterior cingulate and superior temporal cortices). There have been previous efforts to disentangle effects of childhood trauma and psychiatric disorders on brain structure. Overall GM volume, and volumes of regions in the prefrontal cortex, including the anterior cingulate cortex and inferior frontal gyrus, were negatively correlated with sexual abuse severity in individuals with psychotic disorders (Sheffield et al., 2013). Similarly, hippocampal volume was reduced in women with a history of prepubertal physical and/or sexual abuse and current unipolar major depressive disorder (Vythilingam et al., 2002; Frodl et al., 2010; Opel et al., 2014). The amygdala has also been implicated in childhood trauma and mood and anxiety disorders (Nemeroff, 2016). Functional MRI studies found that amygdala responsiveness to threat-related facial expressions was strongly associated with Childhood Trauma Questionnaire (CTQ) scores, an index of childhood adversity, an effect not attributed to recent life stressors, current depression or anxiety or sociodemographic factors (Dannlowski et al., 2012). A meta-analysis of neuroanatomy in children/adolescents and adults with a history of childhood maltreatment and comorbid psychiatric disorders (e.g., anxiety, depression) reporting the prefrontal cortex, hippocampus, parahippocampus, striatum and OFC as regions showing structural alterations associated with childhood trauma (Lim et al., 2014). The most consistent brain structural abnormalities in this meta-analysis were decreases in GM volumes in the right OFC-temporo-limbic and left inferior frontal regions, as attributed to the typically observed deficits in cognitive control in this population (Lim et al., 2014).

Drug addiction is associated with reductions in GM volumes across several of the same regions affected by childhood maltreatment (Hall et al., 2015). Decreased GM density in mesocorticolimbic regions in adults with substance use disorders has been attributed to genetics (Alia-Klein et al., 2011) and factors including the severity of drug use (Sim et al., 2007; Moeller et al., 2016), craving (Wrase et al., 2008; Morales et al., 2015), abstinence (Connolly et al., 2013), or co-morbid depression (Goodkind et al., 2015).

The underlying physiology of decreased GM in substance use disorders may be associated with the acute impact of drugs (as seen with psychostimulants) on inducing large and fast increases in extracellular dopamine, which mimic yet surpass those induced by physiological dopamine cell firing, with marked decreases in dopamine D2 receptor availability and dopamine release with chronic drug abuse (Volkow et al., 2009). This decrease in dopamine function is associated with reduced regional activity in the OFC (Volkow et al., 2009). In addition, in the OFC psychostimulants have been associated with long-lasting decreases in dendritic density (Crombag et al., 2005) likely reflecting decreases in neuronal plasticity (Schoenbaum and Shaham, 2008), which is consistent with decreased GM in the OFC in human substance use disorders (Franklin et al., 2002; Ersche et al., 2013; Hall et al., 2015). Childhood trauma, however, may also be a contributing factor impacting structural and functional deficits in this region in ways that heighten vulnerability to impulsivity, depression symptoms, and substance use disorders in adulthood (Jaffee et al., 2004; Goldstein and Volkow, 2011; McCrory et al., 2011; Puetz and McCrory, 2015). Indeed, childhood adversity enhances the development of antisocial personality traits including reduced constraint, and may lead to chronic depression in adulthood (Beach et al., 2010; Hengartner et al., 2015; Khan et al., 2015; Negele et al., 2015; Thibodeau et al., 2015; Nelson et al., 2017). Low constraint (as a measure of impulsivity) and depression have been identified as risk factors for substance use and are central to the substance use disorders phenotype (Crews and Boettiger, 2009; de Wit, 2009; Green et al., 2012).

Understanding the neural mechanisms that may underlie the impact of childhood maltreatment on substance use disorders could contribute to pre-empting the adverse consequences of exposure and informing neural investigations of addictions, individualized addiction interventions, and to address the long-lasting consequences of childhood trauma exposure (Heilig et al., 2016; Teicher et al., 2016).

The purpose of this study is to examine the potential association between childhood maltreatment and gray matter concentration (GMC) effects in individuals with cocaine use disorder. We further explored, as a *post hoc* analysis, the contribution of the demographic and clinical variables that could contribute to the extent of the observed structural deficits. Since a high incidence of early trauma has been observed in individuals with cocaine use disorders (iCUD; Back et al., 2000; Khoury et al., 2010), and because both substance use disorders and maltreatment are associated with OFC GM deficits, and considering the impact of psychostimulants on OFC neuroplasticity and psychiatric comorbidity, we hypothesized that: (1) iCUD who have history of childhood trauma will exhibit reduced GMC of the OFC; and (2) childhood trauma will explain OFC GM variability in iCUD above and beyond the contribution of demographics, drug effects, and constraint and depression symptoms.

## Materials and Methods

### Participants

Seventy six right-handed individuals were recruited by advertisements in local newspapers and partitioned into three groups: 29 healthy controls (CON), 23 iCUD with low childhood trauma (CUD-L), and 24 iCUD with high childhood trauma (CUD-H). All participants provided written informed consent in accordance and approval of the Institutional Review Board of the Icahn School of Medicine at Mount Sinai. All participants were healthy, were not taking any medications, and were excluded if they had contraindications to the magnetic resonance (MR) imaging environment (e.g., metal in the body or claustrophobia), history of head trauma or loss of consciousness (>30 min), other neurological disease, history of major medical conditions (cardiovascular, endocrinologic, oncologic, or autoimmune diseases), major psychiatric disorders (other than cocaine dependence and alcohol and past cannabis abuse for the CUD group and/or nicotine dependence for both groups), and urine positive (Biopsych; Biopsych Triage, San Diego, CA, USA) for psychoactive drugs or their metabolites (phencyclidine, benzodiazepines, amphetamines, cannabis, opiates, barbiturates and inhalants) except for cocaine in iCUD. With the exception of cocaine, alcohol, cannabis and nicotine, current use or dependence on other drugs was denied. See Table 1 for comparison of demographic, drug use and clinical characteristics between the groups.

**Table 1:** Demographic, drug use and clinical variables.

Characteristics	Controls *N* = 29	Cocaine users low childhood trauma *N* = 23	Cocaine users high childhood trauma *N* = 24
**Demographics**			
Age	41.9 ± 7.9	47.0 ± 7.9	45.8 ± 7.8
Education*	14.6 ± 1.9	13.1 ± 1.8	12.7 ± 1.6
Gender	20 men (69.0%)	20 men (87.0%)	18 men (75.0%)
Race	22 African American (75.9%)	19 African American (82.6%)	16 African American (66.7%)
**Drug use lifetime years/age of initiation/years of heavy use**			
Nicotine	^1^16.7 ± 15.5/^1^16.3 ± 2.2/NA	^2^21.8 ± 11.2/^2^15.4 ± 4.1/NA	^3^22.3 ± 11.9/^3^17.4 ± 6.7/NA
Alcohol	9.3 ± 10.1/17.3 ± 5.8/1.6 ± 1.2	14.8 ± 12.1/15.3 ± 3.6/3.9 ± 2.9	17.3 ± 10.1/13.9 ± 2.8/4.1 ± 3.6
Cocaine	NA	18.5 ± 8.8/20.9 ± 5.5/3.8 ± 3.8	16.8 ± 9.5/20.7 ± 6.3/6.9 ± 7.8
Cannabis**	0.6 ± 2.1/19.0 ± 2.9/0.6 ± 0.3	5.9 ± 7.5/17.2 ± 1.4/1.2 ± 0.5	10.3 ± 12.2/20.1 ± 5.3/1.5 ± 1.7
Severity of cocaine dependence	NA	4.0 ± 5.1	4.8 ± 5.2
Cocaine craving	NA	13.3 ± 10.7	12.8 ± 14.0
Cocaine withdrawal	NA	15.9 ± 13.0	16.1 ± 12.8
Cocaine: days since last use^4^	NA	4.0 ± 4.6	1.7 ± 1.2
Current alcohol	0	2 abuse	11 dependence
**Clinical**			
Childhood trauma^‡^	34.2 ± 5.1	33.2 ± 5.4	57.9 ± 11.1
Constraint^‡^	82.3 ± 8.2	82.6 ± 9.4	^5^71.7 ± 14.1
Depression^†^	2.9 ± 4.2	5.6 ± 6.6	10.1 ± 8.5

Notes: *p < 0.001 (Controls > Cocaine users low = Cocaine users high); **p < 0.001 (Controls < Cocaine users low = Cocaine users high); ^‡^p < 0.001, ^†^p = 0.055 (Controls = Cocaine users low < Cocaine users high; for constraint Controls = Cocaine users low > Cocaine users high); NA: not applicable; ^1^N = 10; ^2^N = 21; ^3^N = 22; ^4^Reported number of days since last cocaine use prior to urine test (N = 7 for each CUD group); ^5^N = 23; The instruments used to derive drugs use/clinical measures: the Structured Clinical Interview for DSM-IV Axis I Disorders (research version; Ventura et al., 1998), the Addiction Severity Index (McLellan et al., 1992), Cocaine Selective Severity Assessment Scale (assessing withdrawal symptoms; Kampman et al., 1998), Severity of Dependence Scale (Gossop et al., 1992), and Cocaine Craving Questionnaire (Tiffany et al., 1993). Childhood Trauma Questionnaire (Bernstein et al., 2003) was used to assess childhood trauma. Beck Depression Inventory-II (BDI; Beck et al., 1996) was used to assess the severity of depressive symptoms occurring over the 2 weeks preceding the study. The Multidimensional Personality Questionnaire (MPQ) Brief Form (Patrick et al., 2002; Tellegen and Waller, 2008) was used as a trait measure of constraint.

### Measures

#### Substance Use and Psychiatric Assessment

The Structured Clinical Interview for *DSM-IV* Axis I Disorders (research version; Ventura et al., 1998), the Addiction Severity Index (McLellan et al., 1992), Cocaine Selective Severity Assessment Scale (assessing withdrawal symptoms; Kampman et al., 1998), Severity of Dependence Scale (Gossop et al., 1992), and Cocaine Craving Questionnaire (Tiffany et al., 1993) were used to assess substance use and psychiatric history.

#### Childhood Trauma Questionnaire

Childhood maltreatment was assessed in all 76 participants with the brief screening version of the CTQ (Bernstein et al., 2003). Due to the high prevalence of co-occurring maltreatment types (Scher et al., 2004), and the association of exposure to multiple types of trauma with poor health outcomes (Huang et al., 2012; Chen et al., 2016; Shalev et al., 2016), we used the CTQ total score, calculated as the sum of scores from each maltreatment subtype, as a continuous measure of individual history of maltreatment (Elton et al., 2014). Following Elton et al. (2014), low vs. high childhood trauma was determined based on a median split of the total CTQ score (= 43; range: 25–125) among all iCUD. Consistent with the median split, the current results showed higher rates of childhood trauma among the CUD-H as compared with the other two study groups and with normative data from a community sample (Scher et al., 2004), where median split of CTQ total scores were = 32 for both sexes combined (Normative data < CUD-H, *t* = 11.4, *p* < 0.001; Normative data = CUD-L, *t* = 1.08, *p* = 0.29; Normative data < CON, *t* = 2.4, *p* = 0.025).

#### Depression Symptoms

Beck Depression Inventory-II (BDI; Beck et al., 1996) was used to assess the severity of depressive symptoms occurring over the 2 weeks preceding the study. The BDI-II is based on the DSM-IV criteria (American Psychiatric Association, 2000), and is considered a valid and reliable instrument for depression screening in the general population (Beck et al., 1996; Vanheule et al., 2008). The items are summed to give a total score (range 0–63). A higher score on the BDI-II denotes more severe depression.

#### Personality

The Multidimensional Personality Questionnaire (MPQ) Brief Form (Patrick et al., 2002; Tellegen and Waller, 2008) was used as a trait measure of constraint. Its scales represent three higher order factors and 10 primary personality dimensions. The higher order factor of constraint (reverse-scored impulsivity and behavioral restraint) embodies both affect and temperament constructs (basic parameters of emotional and behavioral regulation).

### Voxel-Based Morphometry

Voxel based morphometry (VBM), which enables an unbiased voxel-by-voxel comparison of cortical volumes (Whitwell, 2009), was used to quantify regional GMC in study participants.

#### MRI Acquisition and Processing

T1-weighted anatomical images were acquired on a 3T Skyra (Siemens, Erlangen, Germany) using vendor provided 32 channel head coil with a 3D MPRAGE sequence [FOV 256 × 256 × 179 mm^3^, 0.8 mm isotropic resolution, TR/TE/TI = 2400/2.07/1000 ms, flip angle 8° with binomial (1, −1) fat saturation, bandwidth 240 Hz/pixel, echo spacing 7.6 ms, inplane acceleration (GRAPPA) factor of 2, total acquisition time ~7 min]. Structural T1 images were preprocessed using the “HCP PreFreeSurfer structural pipeline” (based on FSL 5.0.6 and FreeSurfer 5.3.0-HCP) to align the origin to the anterior– posterior commissure line, correct image distortions (bias-field inhomogeneities), and to normalize T1-weighted images to the MNI space using a FLIRT affine linear and then a FNIRT nonlinear registration. FSL’s image registration tools were applied to register the native structural scans to the MNI template. FLIRT (FMRIB’s linear image registration tool)^1^ was used for linear (affine; translate, rotate, zoom and shear) registration of images. For instances where the linear transform did not achieve adequate registration, FNIRT (FMRIB’s non-linear image registration tool)^2^ was used for non-linear registration.

#### Voxel-Based Morphometry Analysis

All VBM procedures were conducted using Statistical Parametric Mapping (SPM12 and SPM8; Wellcome Trust Centre for Neuroimaging, UK)^3^, a program running through Mathworks (Matlab 7.5; MathWorks, Natick, MA, USA) with VBM8 toolbox (Gaser, C, University of Jena, Department of Neurology and Psychiatry, Germany)^4^. Total cortical GMC measures were obtained by unified segmentation (Ashburner and Friston, 2005) of each T1 image into GM, white matter and cerebrospinal fluid. A hidden Markov random field was used to maximize segmentation accuracy (Cuadra et al., 2005). The segmentation was then visually inspected for accuracy. Unmodulated normalized GM images (which are GMC, and thereby differ from GM volume that consists of modulated GM images) were smoothed using an 8-mm full-width at half-maximum Gaussian kernel.

Although we had a region specific hypothesis for the OFC, we conducted whole-brain analyses, recognizing that other regions may also be involved (e.g., limbic). We performed a one-way analysis of covariance (ANCOVA) at the whole-brain level for between group comparisons to assess the effect of childhood trauma on GMC with education, age, and total intracranial volume (TIV) included in the models as covariates. We applied a search threshold of *p* < 0.01, adjusted for cluster size, with an extent threshold of 100 contiguous voxels. Only voxels which survived correction for multiple comparisons in the entire volume using the false discovery rate (FDR) method (*p* < 0.05) were considered as significant (Genovese et al., 2002). To further examine the main effect and investigate the contribution of childhood trauma, we conducted a planned follow up comparison between high and low trauma among CUD (CUD-L vs. CUD-H) using whole-brain statistical parametric maps. Here we controlled for multiple comparisons by using the more stringent voxel-level family wise error (FWE) corrected method (*p* < 0.05) at the cluster level. The contribution of childhood trauma to OFC GMC within the entire CUD group (the only region that differed between *CUD-H and CUD-L)* above and beyond that of the other variables (i.e., demographics and cocaine use, that have not been included in the previous steps of analysis) was evaluated by a multiple regression analysis, conducted in SPSS version 23.0 (SPSS Inc., Chicago, IL, USA). This *post hoc* analysis was an additional step to verify that the reported whole brain effects were not driven by other variables that had significant differences between the groups of interest. The first block of this hierarchical regression model included age, education, TIV, and lifetime years of drug use (cocaine, alcohol and cannabis). The second block added constraint and depression, and the third block added the CTQ total scores. To further characterize the potential psychological and behavioral implications of GMC reductions *across the whole sample*, we examined correlations between the GMC variance in the selected region of interest with constraint and depression.

## Results

For descriptive characteristics of the sample see Table 1. The three groups were matched on gender, race and age, but differed in education (CON > CUD-L = CUD-H, *p* < 0.001; used as a covariate in all analyses). Lifetime drug use (in years) of nicotine, alcohol, cocaine, and cannabis, and age of drug use initiation, and years of heavy use (besides nicotine), as well as severity of cocaine dependence, cocaine craving, withdrawal symptoms, time since last cocaine use and current alcohol dependence did not differ between the CUD groups. Yet, iCUD with increased childhood trauma severity reported reduced constraint (CON = CUD-L > CUD-H, *p* < 0.001) and a trend for increased depression symptoms (CON = CUD-L < CUD-H, *p* = 0.055).

**Figure 1:**
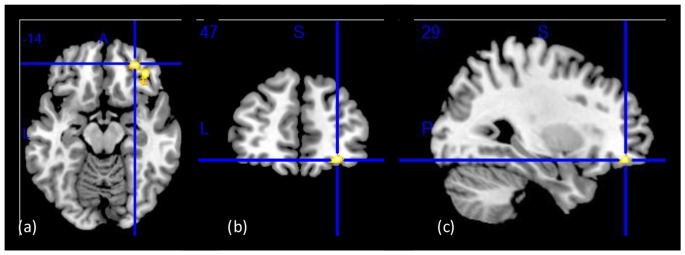
Group main effect on gray matter concentration (GMC). Structural images showing a group main effect (CON > CUD-L > CUD-H) in a whole-brain ANOVA in *the right lateral orbitofrontal cortex (OFC*; BA11 peak-voxel: *x* = 29, *y* = 47, *z* = −14, 883 voxels, cluster-level *p*_FDR–corr_ = 0.02), while controlling for age, education, and total intracranial volume (TIV). **(A)** Axial view; **(B)** coronal view; **(C)** sagittal view of the right hemisphere. The right side of the image corresponds to the right side of the brain. Figure derived with significance level of *p* < 0.001 (uncorrected) to illustrate the group main effect.

**Figure 2:**
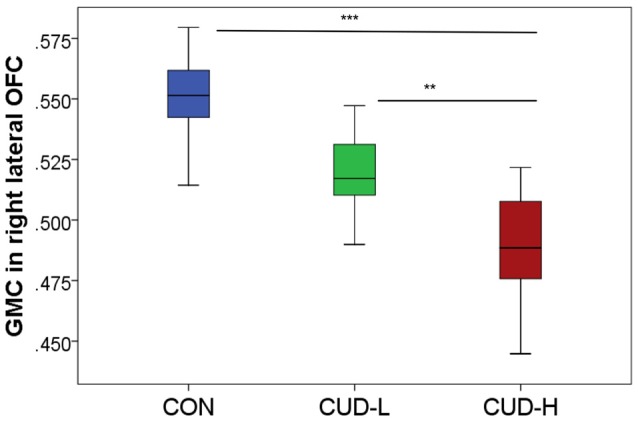
Orbitofrontal gray matter concentration by group. Box-and-whisker plot (error bars representing standard deviation) of estimated cluster concentration by group, showing follow-up between-group comparisons of the main group ANOVA (BA11, peak-voxel: *x* = 29, *y* = 47, *z* = −14, 883 voxels); CON, controls; CUD-L, Individuals with cocaine use disorder with low childhood trauma; CUD-H, Individuals with cocaine use disorder with high childhood trauma; OFC, Orbitofrontal cortex; GMC, Gray matter concentration. **p_FWE-corr_ < 0.05; ***p_FWE-corr_ < 0.001.

The whole-brain SPM analysis showed a significant group main effect in the right lateral OFC, the bilateral middle temporal gyri, and the dorsal anterior cingulate gyrus (Table 2 and Figure 1). Follow-up pairwise whole-brain comparisons showed significantly reduced GMC in the right lateral OFC in the CUD-H as compared to CON and even as compared to CUD-L; In contrast, CUD-L and CON groups did not differ (Table 2 and Figure 2). Additionally, reduced GMC in the bilateral middle temporal gyri were observed in CUD-H as compared to CON. Although there was a main effect in the dorsal anterior cingulate gyrus, the more stringent *post hoc* between group comparisons were not significant. An alcohol dependence diagnosis status was not associated with the GMC results, nor did correlations between each drug use variable in Table 1 (lifetime years, age initiation and years of heavy use) with GMC in our main effect regions reached significance.

**Table 2:** Main effect and between group comparisons of whole-brain gray matter concentration.

Region	BA	Side	Voxels	Peak *Z*	*Cluster P* Value	*x*	*y*	*z*
**Group main effect**^†^
Lateral orbitofrontal gyrus	11	R	883	4.05	0.020	29	47	−14
Middle temporal gyrus	21	R	1069	3.64	0.014	59	3	−27
Middle temporal gyrus	22	L	1010	3.86	0.014	−63	−15	−8
Dorsal anterior cingulate gyrus	32	L	1108	3.55	0.014	−11	33	19
**Between-groups comparisons**^‡^
**CUD-L > CUD-H**
Lateral orbitofrontal gyrus	11	R	1537	3.49	0.035	30	48	−12
**CON > CUD-H**
Lateral orbitofrontal gyrus	47	R	15,097	4.53	<0.001	39	41	−15
Middle temporal gyrus	21	R		4.20		59	3	−27
Middle temporal gyrus	22	L	4802	4.31	<0.001	−63	−15	−8

*Note: ^†^Analyses are one-way ANCOVAs (controlling for age, education and total intracranial volume), thresholded at p < 0.01, uncorrected, and an extent threshold of 100 contiguous voxels. Only voxels which survived correction for multiple comparisons in the entire volume using the false discovery rate (FDR) method, p < 0.05, were considered significant; ^‡^Analyses are pairwise between-groups comparisons of gray matter concentration (controlling for education, age, and total intracranial volume), corrected for multiple comparisons using the voxel-level family wise error (FWE) corrected method, p < 0.05; CON, controls; CUD-L, Individuals with cocaine use disorder with low childhood trauma; CUD-H, Individuals with cocaine use disorder with high childhood trauma; BA, Brodmann Area; R, right; L, left*.

**Figure 3:**
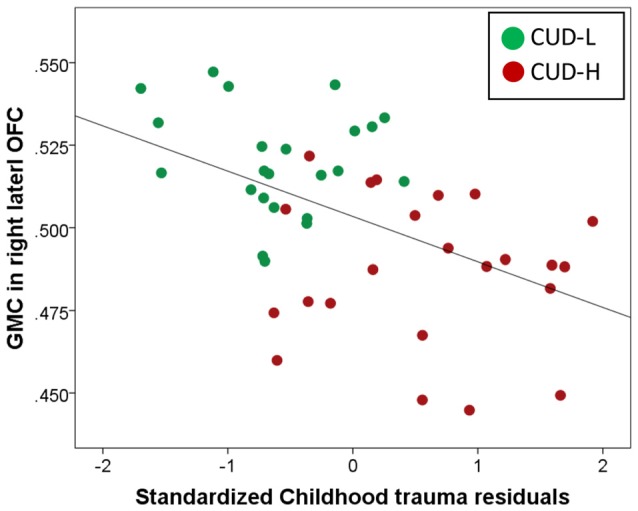
Unique childhood trauma effects on gray matter concentration of the orbitofrontal cortex in individuals with cocaine use disorder. Scatter plot of regression of gray matter concentration (GMC; extracted from BA11, peak-voxel: *x* = 29, *y* = 47, *z* = −14, 883 voxels) with standardized childhood trauma residuals across all individuals with cocaine use disorder (Childhood Trauma Questionnaire, CTQ total while controlling for age, total intracranial volume, education, lifetime drug use, and constraint and depression). Childhood maltreatment was assessed with the CTQ (Bernstein et al., 2003); OFC, Orbitofrontal cortex; CUD-L, Individuals with cocaine use disorder with low childhood trauma; CUD-H, Individuals with cocaine use disorder with high childhood trauma.

Across all CUD, the *post hoc* analysis using hierarchical regression demonstrated that the continuous measure of childhood trauma accounted for 37.7% of variance in GMC in the right lateral OFC (third block; Δ*R*^2^ = 0.24, *p* < 0.001; Table 3), while constraint and depression explained 10.2% (second block; Δ*R*^2^ = 0.20, *p* = 0.012), above and beyond the effects of age, education, TIV and lifetime drug use variables (first block *p* = 0.853). Thus, beyond demographics and drug use, and beyond constraint and depression, the experience of childhood trauma related to OFC GMC reduction (Figure 3).

**Table 3:** The contribution of age, education, drug use, constraint, depression and childhood trauma to orbitofrontal gray matter concentration (extracted from BA11, peak-voxel: *x* = 29, *y* = 47, *z* = −14, 883 voxels) in individuals with cocaine use disorder.

Multiple regression	Block 1			Block 2			Block 3		
Variable	*B*	*SE B*	*β*	*B*	*SE B*	*β*	*B*	*SE B*	*β*
Age	−0.001	0.001	−0.212	−0.001	0.001	−0.192	−0.001	0.000	−0.291*
Education	0.000	0.002	0.010	−0.002	0.002	−0.131	−0.001	0.002	−0.099
TIV	−2.172*E* − 5	0.000	−0.119	−2.047*E* − 5	0.000	−0.112	−1.364*E*−5	0.000	−0.075
**Drug use lifetime**									
Cocaine	0.001	0.000	0.190	0.000	0.000	0.161	0.000	0.000	0.124
Alcohol	9.441*E*−6	0.000	0.004	2.132*E*−5	0.000	0.009	0.000	0.000	0.080
Cannabis	1.816*E*−5	0.000	0.007	0.000	0.000	0.096	0.000	0.000	0.163
Constraint				0.001	0.000	0.367*	0.000	0.000	0.101
Depression				−0.001	0.000	−0.287	0.000	0.000	−0.123
Childhood trauma^†^							−0.001	0.000	**−0.607**
Adjusted *R*^2^		−0.082			**0.102**			**0.377**	
*F* for change in *R*^2^		0.431			5.007*			17.301**	

Note: *p < 0.05; **p < 0.001; ^†^Childhood trauma entered here as a continuous variable of total score; total intracranial volume (TIV); Beck Depression Inventory-II (BDI; Beck et al., 1996) was used to assess the severity of depressive symptoms occurring over the 2 weeks preceding the study; The Multidimensional Personality Questionnaire (MPQ) Brief Form (Patrick et al., 2002; Tellegen and Waller, 2008) was used as a trait measure of constraint; Childhood maltreatment was assessed with the Childhood Trauma Questionnaire (CTQ; Bernstein et al., 2003).

**Figure 4:**
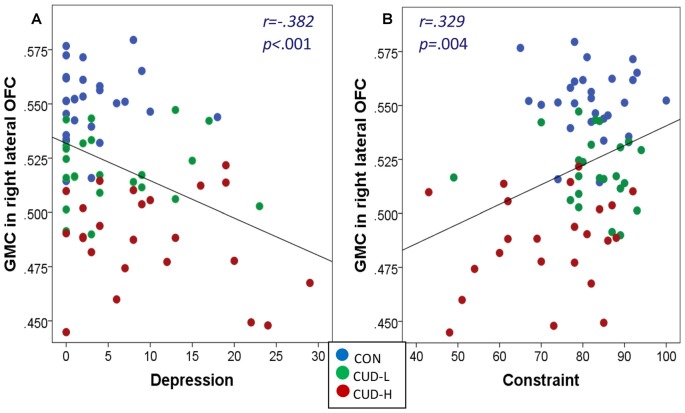
**(A,B)** Gray matter concentration of orbitofrontal cortex associations with depression and constraint. Scatter plots of correlations of gray matter concentration (GMC; BA11, peak-voxel: *x* = 29, *y* = 47, *z* = −14, 883 voxels) with depression and constraint across the whole sample revealed that: (a) the greater the depression the lower the GMC (*r* = −0.382, *p* = 0.001); and (b) the lower the constraint (*r* = 0.329, *p* = 0.004) the lower the GMC of the right lateral orbitofrontal cortex (OFC); Beck Depression Inventory-II (BDI; Beck et al., 1996) was used to assess the severity of depressive symptoms occurring over the 2 weeks preceding the study; The Multidimensional Personality Questionnaire (MPQ) Brief Form (Patrick et al., 2002; Tellegen and Waller, 2008) was used as a trait measure of constraint; CON, controls; CUD-L, Individuals with cocaine use disorder with low childhood trauma; CUD-H, Individuals with cocaine use disorder with high childhood trauma.

Correlations of GMC across the whole sample revealed that the decreased GMC in the right lateral OFC was also significantly associated with increased depression (*r* = −0.382, *p* = 0.001) and decreased constraint (*r* = 0.329, *p* = 0.004; Figure 4).

## Discussion

This study was conducted to discern the morphological and comorbid symptom properties of childhood maltreatment in iCUD. Consistent with our hypotheses, the main results of this study are that: (1) GMC of the right lateral OFC is reduced with greater childhood trauma in iCUD such that the most abnormality was observed in iCUD with high trauma, followed by iCUD with low trauma, and then by controls; (2) above and beyond the effects of demographics, lifetime drug use, constraint and depression, childhood trauma has a noticeable and significant effect on OFC GMC reduction in iCUD.

Our main results are consistent with previous findings demonstrating brain’s GM decreases as related to childhood trauma in individuals with a history of childhood maltreatment and comorbid psychiatric disorders. For example, an extensive meta-analysis (comprising 331 individuals with a history of childhood maltreatment and psychiatric comorbidities and 362 comparison subjects) showed that deficits in the right OFC/superior temporal gyrus were the most robust GM abnormalities in non-addicted individuals (Lim et al., 2014). Reduced GMC in the middle temporal gyrus was observed in CUD-H as compared to CON (but not CUD-L) as well as in subjects with childhood verbal aggression trauma measured in early adulthood (Tomoda et al., 2011). Our main results are also consistent with a morphometric analysis of 148 healthy individuals (without any history of psychiatric disorders) where high CTQ scores were associated with reduced GM volumes in the OFC and anterior cingulate gyrus (Dannlowski et al., 2012). In contrast to this latter study, we did not report significant results for the hippocampus, insula, and caudate. Of note is that in a previous study of addicted individuals as compared to controls, childhood trauma was similarly associated with GMC reductions in the hippocampus (and also in the parahippocampus and anterior fusiform gyrus) in individuals with alcohol (95.8% of the sample), cocaine (79.2%), and/or cannabis (77.8%) use disorders (Van Dam et al., 2014). These inconsistencies in results may be attributed to differences in the samples and methods used in these studies. Although smaller in size (our total sample size was 76 vs. 148 and 177 in the previous studies), we only included current actively drug using iCUD with nearly double lifetime years of cocaine use for our participants as compared to the subjects included in the previous study in addiction (Van Dam), representing a pattern of chronic and severe use of cocaine. We also excluded for psychiatric comorbidity (as opposed to comorbidity of post-traumatic stress disorder and anxiety in the Van Dam study) and analyzed GMC (vs. GM volume in the previous studies). Finally, the present study also examined childhood trauma using continuous CTQ scores, whereas the Van Dam study approached CTQ as a dichotomous measure; although the Dannlowski study also used the CTQ as a continuous measure, the extent of childhood trauma was qualitatively different between our studies (with mean CTQ scores of 33.4 ± 10.0 for the previous study, which is similar to the scores of our CON and CUD-L groups but not the CUD-H group). Another expected difference with the Dannlowski study was in age, where the mean age of participants was over a decade younger than participants in the current sample. These variations highlight the importance of carefully considering variability in sample characteristics (including years since trauma occurred and psychiatric comorbidity) and study methods (e.g., GM concentration vs. volume) when comparing between morphometric studies of effects of childhood trauma.

The current findings indicate that, above and beyond depression and constraint, major psychological constructs that are part of the addiction phenotype, childhood maltreatment had an apparent effect on GMC in the OFC in iCUD. In the addiction model of iRISA (Goldstein and Volkow, 2002, 2011), the OFC (and anterior cingulated gyrus as well as other prefrontal regions) has been implicated in motivation (e.g., enhanced motivation to procure drugs but decreased motivation for other goals), awareness and interoception, learning and memory, decision making (e.g., coding reinforcers) and salience attribution (e.g., affective value appraisal; Goldstein and Volkow, 2002, 2011). It is possible that prefrontal cortical GM volumes, which mature later during childhood and adolescence (Sowell et al., 2003) at a vulnerable developmental timing that coincides with drug experimentation, predispose individuals to substance abuse risk (Weiland et al., 2014). It is also possible that prenatal drug exposure (to alcohol and/or cigarette smoking), which are associated with reduced OFC cortical thickness, increases substance use during adolescence (Kühn et al., 2016). Both factors may be more pronounced with childhood maltreatment.

Previous research showed that childhood maltreatment contributes to a high prevalence of co-morbid personality disorders in addicted populations (Bernstein et al., 1998). Indeed, in our study CUD-H reported less constraint and higher depression than CUD-L. Such greater symptomatology in CUD-H may confer a greater severity of substance use disorders and an increased risk for relapse, as associated with lower GMC in the OFC. Thus, iCUD with histories of childhood trauma may represent a clinically and biologically distinct subtype. Recognition of this distinction may be essential in determining the biological bases of this subtype and guide individualized treatment (Nanni et al., 2012; Teicher and Samson, 2013; Teicher et al., 2016).

These results should be considered in light of the study’s limitations. A major limitation is in absence of a control group who experienced childhood trauma (although note that our healthy control group reported higher levels of childhood trauma than the normative CTQ data). Our three group study design is similar to previous studies that compared brain’s GM following early life adversity in two groups with psychiatric comorbidity (e.g., major depression and psychotic disorder patients) with and without early trauma and a healthy control group without trauma (e.g., Vythilingam et al., 2002; Sheffield et al., 2013). Nevertheless, future studies including a control group with high childhood trauma and no substance use disorder would be critical to attribute the observed low OFC GMC results specifically to the trauma or to an interaction of trauma with substance use. As well, it remains to be studied whether individuals with childhood trauma with comorbid substance use disorders and major depression represent a qualitatively different subtype than those subjects included in the current study. In addition, given that strong statistical effects were not anticipated (since we were measuring effects of events that probably have occurred decades ago), we used a search threshold of 0.01 uncorrected together with different multiple correction methods (FDR and FWE). Although these analytical strategies enabled us to capture the effects of maltreatment that contributed evident variance beyond demographics and drug use, and comorbid symptomatology, recent reports point to inaccuracy of FDR correction methods for voxel-wise analyses as compared with topological FWE or FDR control (Chumbley et al., 2010); therefore, these results need to be replicated in a larger sample and with more stringent statistical thresholds. Lastly, childhood trauma was retrospectively assessed and self-reported. This approach is broadly accepted as reliable and valid (Dill et al., 1991; Hardt and Rutter, 2004; Anda et al., 2006); nevertheless, future prospective and well documented assessments need to account for type, timing and duration of childhood trauma, since specific neurobiological consequences of extent of childhood trauma have been associated with unique windows of vulnerability in various brain regions (Teicher et al., 2006; Andersen et al., 2008; Teicher and Samson, 2013; Pechtel et al., 2014; Nemeroff, 2016).

## Conclusion

Above and beyond effects of demographics, lifetime drug use and constraint and depression, our results point to a noticeable and significant contribution of adverse childhood experiences, predating drug use, to GMC reduction of the OFC in cocaine use disorders. These findings suggest a link between premorbid environmental stress and OFC morphology in addicted individuals, and highlight the importance of accounting for childhood trauma in future examination of neuroanatomy in substance use. Early trauma may result in a different addiction-related phenotype, due to the potency of the effects of maltreatment on the developing brain. Although MRI scans are not routinely used in treatment, these findings underscore individual variance within the population of substance users and highlight the importance of individualized interventions to ameliorate the possible consequences of childhood trauma exposure for addicted individuals.

## Ethics Statement

This study was carried out in accordance with the recommendations of the Institutional Review Board of the Icahn School of Medicine at Mount Sinai with written informed consent from all subjects. All subjects gave written informed consent in accordance with the Declaration of Helsinki. The protocol was approved by the Institutional Review Board of the Icahn School of Medicine at Mount Sinai.

## Author Contributions

KB, MAP, SJM, GG, AZ, RZG and NA-K (all authors) fulfilled the following four criteria: (1) substantial contributions to the conception or design of the work; or the acquisition, analysis, or interpretation of data for the work; (2) drafting the work or revising it critically for important intellectual content; (3) final approval of the version to be published; and (4) agreement to be accountable for all aspects of the work in ensuring that questions related to the accuracy or integrity of any part of the work are appropriately investigated and resolved.

## Conflict of Interest Statement

The authors declare that the research was conducted in the absence of any commercial or financial relationships that could be construed as a potential conflict of interest.

## Footnotes

^1^http://fsl.fmrib.ox.ac.uk/fsl/flirt/

^2^https://fsl.fmrib.ox.ac.uk/fsl/fslwiki/FNIRT

^3^www.fil.ion.ucl.ac.uk/spm

^4^http://www.neuro.uni-jena.de/vbm/
